# Schisandrin B regulates MC3T3-E1 subclone 14 cells proliferation and differentiation through BMP2-SMADs-RUNX2-SP7 signaling axis

**DOI:** 10.1038/s41598-020-71564-z

**Published:** 2020-09-02

**Authors:** Xueni Wang, Xiuling Liao, Yimin Zhang, Linyao Wei, Yuzhou Pang

**Affiliations:** 1 Guangxi Zhuang Yao Medicine Center of Engineering and Technology, Guangxi University of Chinese Medicine, 13 Wuhe Road, Qingxiu District, Nanning, 530200 China; 2 Guangxi Key Laboratory of Zhuang and Yao Ethnic Medicine, Guangxi University of Chinese Medicine, Nanning, 530200 China; 3 Faculty of Pharmacy, Guangxi University of Chinese Medicine, 13 Wuhe Road, Qingxiu District, Nanning, 530200 China; 4 Guangxi Key Laboratory of Efficacy Study on Chinese Materia Medica, Guangxi University of Chinese Medicine, 13 Wuhe Road, Qingxiu District, Nanning, 530200 China

**Keywords:** Drug discovery, Molecular medicine

## Abstract

Schisandrin B (SchB) is the highest content of biphenyl cyclooctene lignans in *Schisandra chinensis*. It has been reported to have a variety of pharmacological effects, including anti-inflammatory, anti-oxidant, anti-cancer, heart protection, liver protection. In this study, we found that SchB can promote the proliferation of MC3T3-E1 subclone 14 cells. Meanwhile, we found that SchB can regulate the BMP2-SMADs signaling pathway by increasing gene and protein expression of those relative biomolecules. Furthermore, SchB can raise the RUNX2 and SP7 expression in both mRNA and protein levels. Since the role of BMP2-SMADs-RUNX2-SP7 signaling axis in osteoblast proliferation and differentiation has been well documented. The present experimental findings indicate that SchB could promote the proliferation and differentiation of osteoblasts through BMP2-SMADs-RUNX2-SP7 signaling axis.

## Introduction

Schisandrin B (SchB) is one of the lignans with high content from *Schisandra chinensis*^1^. SchB has been reported to have a variety of pharmacological activities, including anti-inflammation^2^, antioxidation^3^, anti-cancer^4,5^ etc. It is worth noting that SchB has been reported to ameliorate chondrocytes inflammation and osteoarthritis by inhibiting NF-κB and MAPK signaling pathways^6^. These findings suggest that SchB may play a potential role in alleviate bone disease. However, the effect of SchB on osteoblasts is unknown.

MC3T3-E1 subclone 14 cells are often used to study the proliferation and differentiation of osteoblasts^7–9^. It is well known BMP2-SMADs signaling pathway play a key role in mediating osteoblast differentiation and osteogenesis^10^. While Runx2 and SP7 are transcription factors that are essential for osteoblast proliferation and differentiation^11,12^.

Here, the effects of SchB on the proliferation of MC3T3-E1 subclone 14 cells were evaluated by MTT assay, and the effects of SchB on the expression of genes and proteins related to BMP2-SMADs-RUNX2-SP7 signaling axis were investigated by quantitative PCR and western blot, respectively.

## Results

### SchB promotes MC3T3-E1 subclone 14 cells proliferation in a certain concentration range

**Figure 1:**
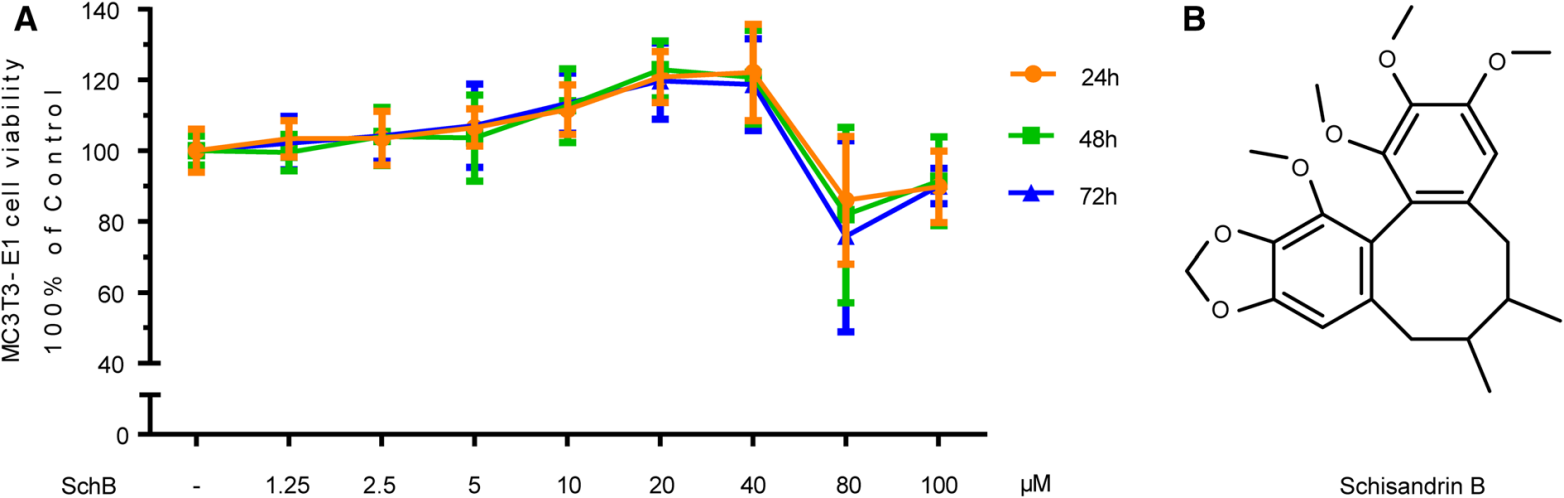
SchB promotes MC3T3-E1 subclone 14 cells proliferation in a certain concentration range. (**A**) Viability of MC3T3-E1 subclone 14 cells treated with different concentrations of SchB; (**B**) Chemical formula of SchB.

Osteoblasts are responsible for bone synthesis, remodeling and healing^13^. Osteoblast proliferation plays an important role in bone maintenance and development. In the present study, SchB stimulated the proliferation of MC3T3-E1 subclone 14 cells in gradually increase degrees at the concentration of 1.25–40 μM, while showed cytotoxicity at concentrations of 80 μM and 100 μM. In addition, after 24 h of treatment, the effect of SchB on the proliferation of MC3T3-E1 cells tended to be flat, and the intensity of action did not increase significantly with the extension of time (Fig. 1).

### SchB promotes BMP2, SMADs and Runx2 gene expression

**Figure 2:**
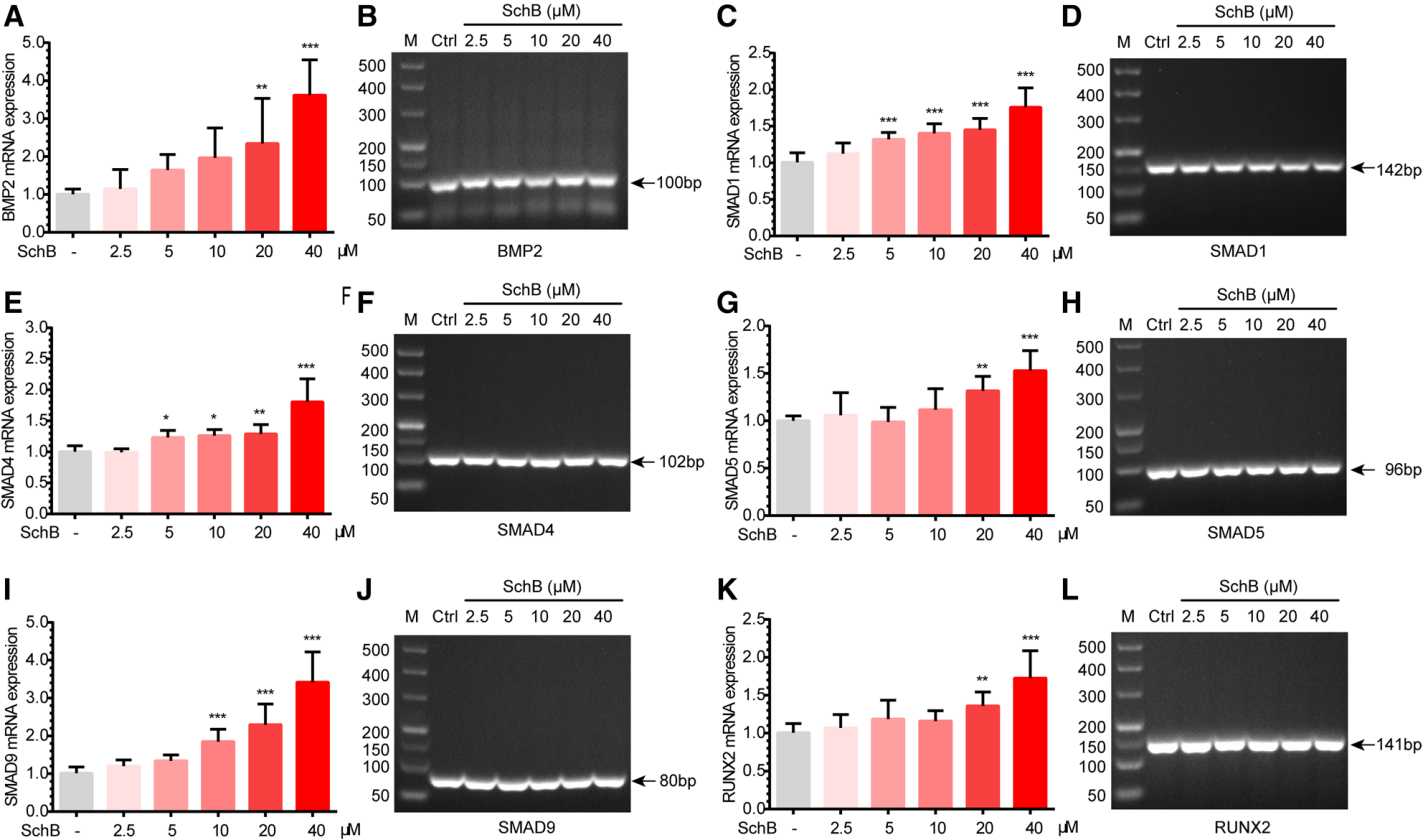
SchB promotes BMP2-SMADs gene expression in MC3T3-E1 subclone 14 cells. (**A**) BMP2 mRNA expression; (**B**) qPCR product length of BMP2; (**C**) SMAD1 mRNA expression; (**D**) qPCR product length of SMAD1; (**E**) SMAD4 mRNA expression; (**F**) qPCR product length of SMAD4; (**G**) SMAD5 mRNA expression; (**H**) qPCR product length of SMAD5; (**I**)SMAD9 mRNA expression; (**J**) qPCR product length of SMAD9; (**K**) RUNX2 mRNA expression ; (**L**) qPCR product length of RUNX2.

The positive correlation between BMP2, SMADs, RUNX2, and osteoblast proliferation and differentiation has been clearly elucidated^11,14^. At first, we found SchB could stimulate the proliferation of MC3T3-E1 subclone 14 cells by the MTT assay. Then attributing to the qPCR test we have confirmed that SchB could promote the expression of BMP2 gene in the concentration range of 5–40 μM, especially at the concentrations of 20 μM and 40 μM. In addition, similar findings have been found in the detection of SMAD1,4,5,9 and RUNX2 gene expression (Fig. 2).

### SchB promotes BMP2, SMADs and Runx2 protein expression

**Figure 3:**
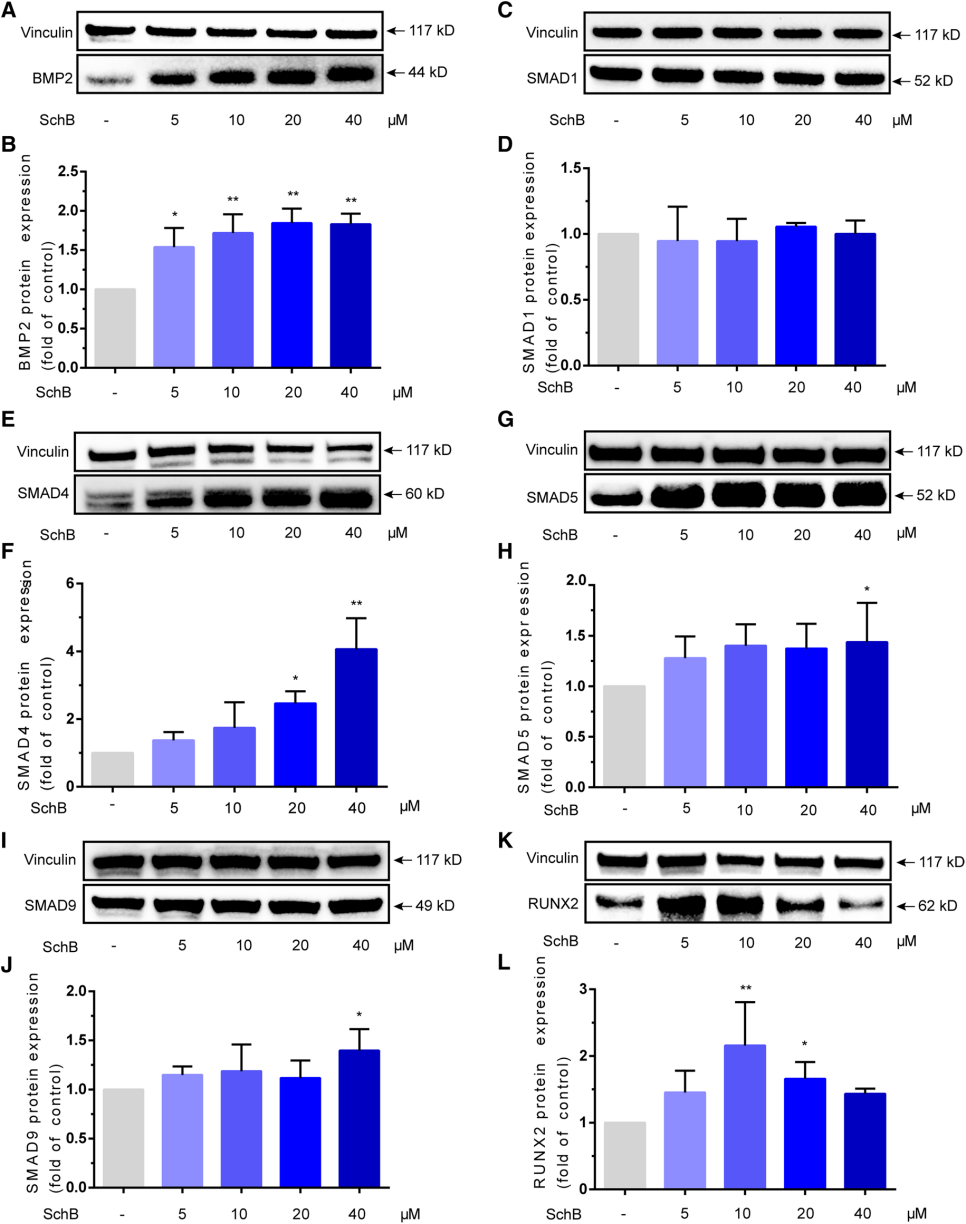
SchB promotes BMP2-SMADs protein expression in MC3T3-E1 subclone 14 cells. (**A**,**B**) Protein expression of BMP2; (**C**,**D**) Protein expression of SMAD1; (**E**,**F**) Protein expression of SMAD4; (**G**,**H**) Protein expression of SMAD5; (**I**,**J**) Protein expression of SMAD9; (**K**,**L**) Protein expression of RUNX2.

Based on the findings at the mRNA level, we further examined the effects of SchB on these molecules at the protein level. Given that 2.5 μM SchB has no significant effect on BMP2-SMADs at the mRNA level, we chose 5–40 μM for protein level detection. The data showed that SchB could promote the protein expression of BMP2 in the concentration range of 5–40 μM. Meanwhile, SchB can significantly up-regulate the expression of SMAD4 protein only at the concentration of 20 and 40 μM, and up-regulate the expression of SMAD5,9 protein at the concentration of 40 μM. However, there was no significant change in the protein expression of SMAD1 (Fig. 3).

### SchB promotes SP7 mRNA and protein expression

**Figure 4:**
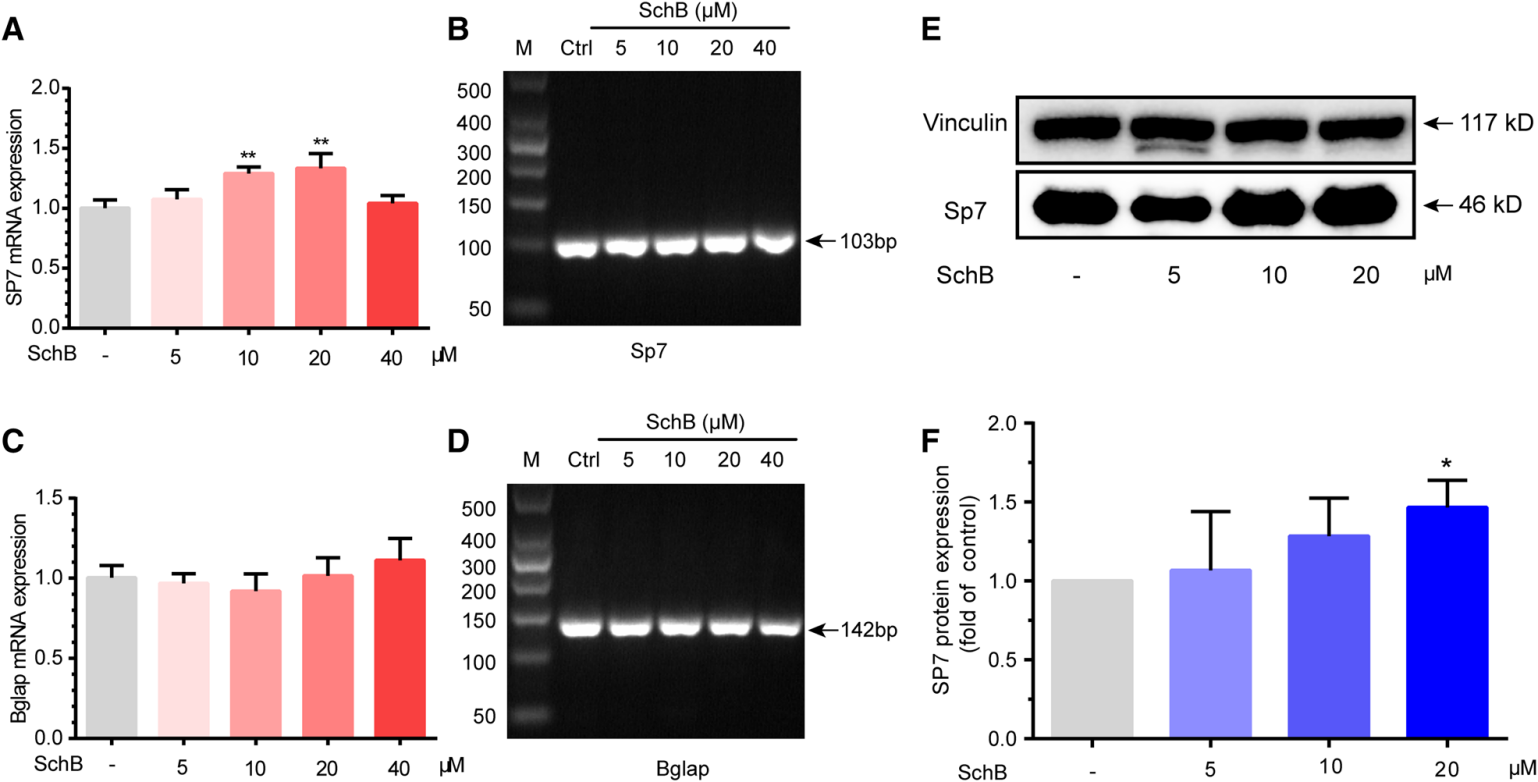
SchB promotes SP7 mRNA and protein expression  in MC3T3-E1 subclone 14 cells. (**A**) mRNA expression of Sp7; (**B**) RT-qPCR product length of Sp7; (**C**) mRNA expression of Bglap; (**D**) RT-qPCR product length of Bglap; (**E**,**F**) Protein expression of SP7.

Sp7 is an essential transcription factor for osteoblast differentiation, which induced by Runx2^12^. Bglap also known as osteocalcin, is an osteoblast marker. Sp7 directly regulates the expression of Bglap through Sp7-binding sites on the promoter region of the gene^15^. In the present study, we observed that SchB could upregulate the mRNA and protein expression of Sp7 while show no significant effect on Bglap (Fig. 4).

## Discussion

In this study, we found that SchB can promote the proliferation of MC3T3-E1 subclone 14 cells and up-regulate the gene and protein expression of biomolecules in BMP2-SMADS signaling pathway. Firstly, SchB can promote the proliferation of MC3T3-E1 subclone 14 cells at the concentration of 1.25–40 μM. However, we note that extending the treating time of SchB does not enhance its effectiveness. This data indicated that SchB can achieve its strongest effect within 24 h. At the level of mRNA, SchB can promote the expression of BMP2, SMADs, Runx2, Sp7 genes a dose-dependent manner but have no impact on Bglap. The expression of BMP2, SMADs, Runx2, SP7 protein was up-regulated to varying degrees, but only SMAD4 and SP7 protein showed a dose-dependent relationship, while the expression of SMAD1 protein had no significant change. These results indicate that SchB may have a high selectivity for SMAD4 and SP7.

In addition, we noticed that 10–20 μM SchB markedly up-regulated the expression of Runx2, while 40 μM SchB had no significant effect on the protein expression of RUNX2 (Fig. 3K, L). Then we found 40 μM SchB also have no impact on mRNA expression of Sp7 (Fig. 4A). Given that SP7 induced by Runx2, this finding is reasonable. However, these series of results suggest that 40 μM SchB may be too high for MC3T3-E1 subclone 14 cells.

**Figure 5:**
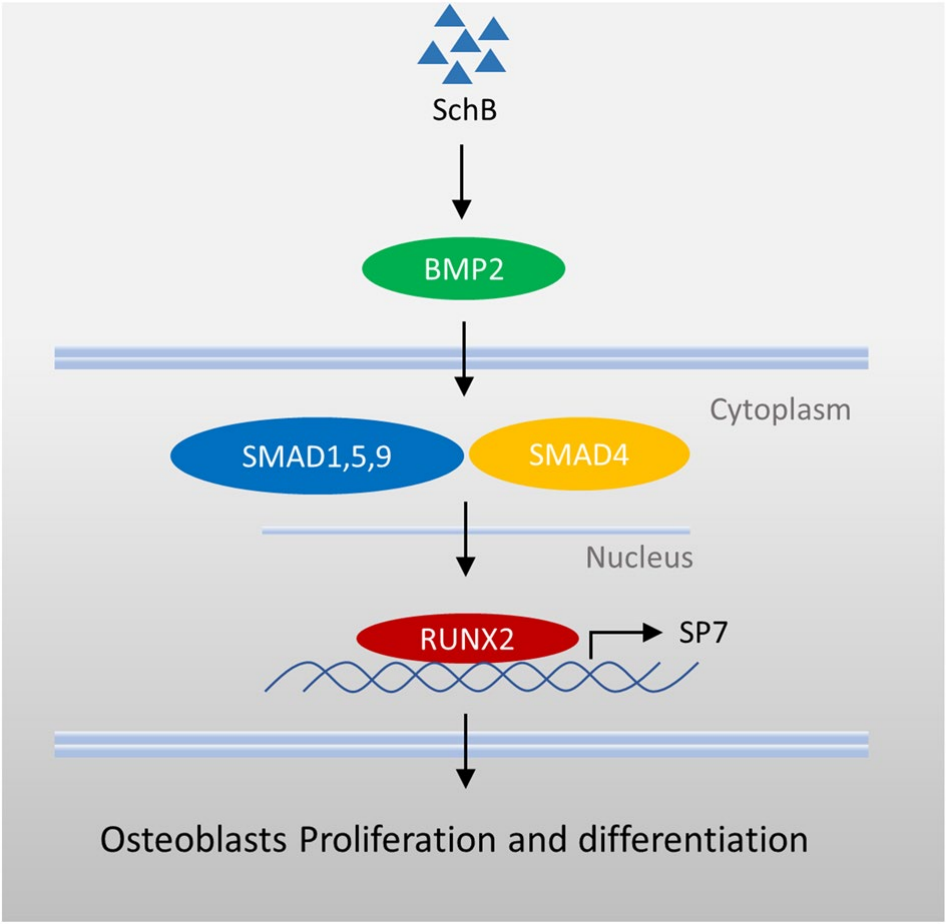
Overview of the mechanism of action of SchB.

Taken together, our findings reveal the effect and mechanism of SchB on MC3T3-E1 subclone 14 cells (Fig. 5), and further confirm its potential value in the treatment of bone-related diseases, especially osteoarthritis and rheumatoid arthritis, which are closely related to inflammation and osteoblasts. Based on the findings of this study, we will further examine the potential role of SchB in the treatment of osteoarthritis and rheumatoid arthritis in vitro and in vivo.

## Materials and methods

### Reagents and antibodies

SchB was purchased from aladdin (Shanghai, China). MEM-α was purchased from Gibco (Beijing, China). Fetal Bovine Serum was from Gemini bio-products (USA). Trypsin-EDTA solution and DMSO were from Solarbio (Beijing, China). Penicillin–Streptomycin solution was from HyClone (USA). TrIzol Reagent, RevertAid First Strand cDNA Systhesis kit and PowerUp SYBR Green Master Mix were purchased from Thermo Fisher Scientific (USA). RIPA buffer and PMSF were purchased from Solarbio (Beijing, China). BMP2 (Cat#18933-1), SMAD1 (Cat#10429-1-AP), SMAD4 (Cat#10231-1-AP), SMAD5 (Cat#12167-1-AP), SMAD9 (Cat#16397-1-AP) rabbit polyclonal antibody, HRP-conjugated Affinipure Goat Anti-Rabbit IgG(H + L) and HRP-conjugated Affinipure Goat Anti-Mouse IgG(H + L) Antibody were purchased from proteintech. RUNX2 (D1L7F) Rabbit mAb was purchased from cell signaling technology (Cat#12556). SP7 Polyclonal antibody was purchased from Invitrogen (Cat#PA5-40509). Chemiluminescent HRP substrate was purchased from Millipore.

### Cell culture

MC3T3-E1 subclone 14 cell line obtained from the committee of type culture collection of Chinese Academy of sciences (Shanghai, China). It was maintained in MEM-α supplemented with 10% (v/v) fetal bovine serum (FBS), 100 units/mL penicillin, and 100 μg/μL streptomycin.

### MTT assay

MTT assay was performed in 96-well plates in sextuplicate. MC3T3-E1 subclone 14 cells were seeded at a density of 5 × 10^3^ cells/well overnight, and treated with compounds for 24 h, 48 h, 72 h respectively. OD490 values of compounds were detected using the Epoch 2 Microplate Spectrophotometer from BioTek Instruments.

### Endogenous gene expression

MC3T3-E1 subclone 14 cells were seeded into 60 mm dishes and grown for 24 h in medium containing 10% FBS. Cells were then treated with DMSO or SchB with the indicated concentrations for 24 h. RNA was extracted and purified using the TrIzol Reagent. cDNA was prepared from 1 μg of RNA with the RevertAid First Strand cDNA Systhesis kit. Diluted cDNA was used to perform qPCR using SYBR Green (Light cycler 96, Roche) with ACTB as the internal standard. Primers for quantitative RT-PCR were listed in Table 1.

**Table 1:** List of primer sequences for qPCR.

Species	Primer code	Primer sequences (5′–3′)	Product length (bp)
Mouse	ACTB fwd	GTGCTATGTTGCTCTAGACTTCG	174
	ACTB rev	ATGCCACAGGATTCCATACC	
Mouse	BMP2 fwd	GAATGACTGGATCGTGGCACCTC	100
	BMP2 rev	GGCATGGTTAGTGGAGTTCAGGTG	
Mouse	Smad1 fwd	TCACAGATCCGTCCAACAATAAGAACC	142
	Smad1 rev	TCCGCATACACCTCTCCTCCAAC	
Mouse	Smad5 fwd	TCTTACCTCCAGTATTAGTGCCTCGTC	96
	Smad5 rev	TGTGCGGTTCATTGTGGCTCAG	
Mouse	Smad8/9 fwd	GGTGTATGCCGAGTGCGTGAG	80
	Smad8/9 rev	CTGGGTGGAAGCCGTGTTGATAG	
Mouse	Smad4 fwd	TGTTGTGACTGTGGATGGCTATGTG	102
	Smad4 rev	CTCGCTCTCTCAATCGCTTCTGTC	
Mouse	RUNX2 fwd	CATGGTGGAGATCATCGCGG	141
	RUNX2 rev	ACCTCTCCGAGGGCTACAAC	
Mouse	Sp7 fwd	GCAGGCATCCACGCAGGCATCTC	103
	Sp7 rev	CCTGGCCCTGACCACCACCTAGC	
Mouse	Bglap	CCCTGGCTGCGCTCTGTCTCTCTG	142
	Bglap	GGGCTGGGGACTGAGGCTCCAAG	

### Western blot

MC3T3-E1 subclone 14 cells were seeded into 60 mm dishes and grown for 24 h in medium containing 10% FBS. Then cells received fresh medium containing the indicated treatments. Whole cell extracts were prepared after 24 h of treatment using RIPA buffer supplemented with 1 mM PMSF. 40 μg of protein per lane was analyzed on 10% SDS-PAGE gels and transferred to PVDF transfer membranes. BMP2, SMADs, Runx2, SP7 protein was detected using antibodies listed above. Images were captured using the Chemidoc CD Touch (Bio-Rad, USA), and images analyzing and processing using the Image Lab 6.0 (Bio-Rad, Chinese edition).

### Statistical analysis

All results were presented as mean ± standard deviation (SD). Statistical significance was determined with One-Way ANOVA. *p* < 0.05 was considered statistically significant.

## Supplementary information

Supplementary Information.
