# Supplementary material for: Schisandrin B regulates MC3T3-E1 subclone 14 cells proliferation and differentiation through BMP2-SMADs-RUNX2-SP7 signaling axis
Source: Sci Rep. 2020 Sep 2;10:14476. doi: 10.1038/s41598-020-71564-z (PMC7468146; doi:10.1038/s41598-020-71564-z)
Supplement: Supplementary file 1 — Supplementary Information. [file 41598_2020_71564_MOESM1_ESM.docx]

**Schisandrin B regulates MC3T3-E1 Subclone 14 cells proliferation and differentiation through BMP2-SMADs-RUNX2-SP7 signaling axis**

Xueni Wang^a,b*^, Xiuling Liao^a,c^, Yimin Zhang^a^, Linyao Wei^d^, Yuzhou Pang^a^

Supplementary Material

1.Fig.3A&B in our manuscript represents the analysis outcomes of the whole uncropped images in Sup.Fig.1.


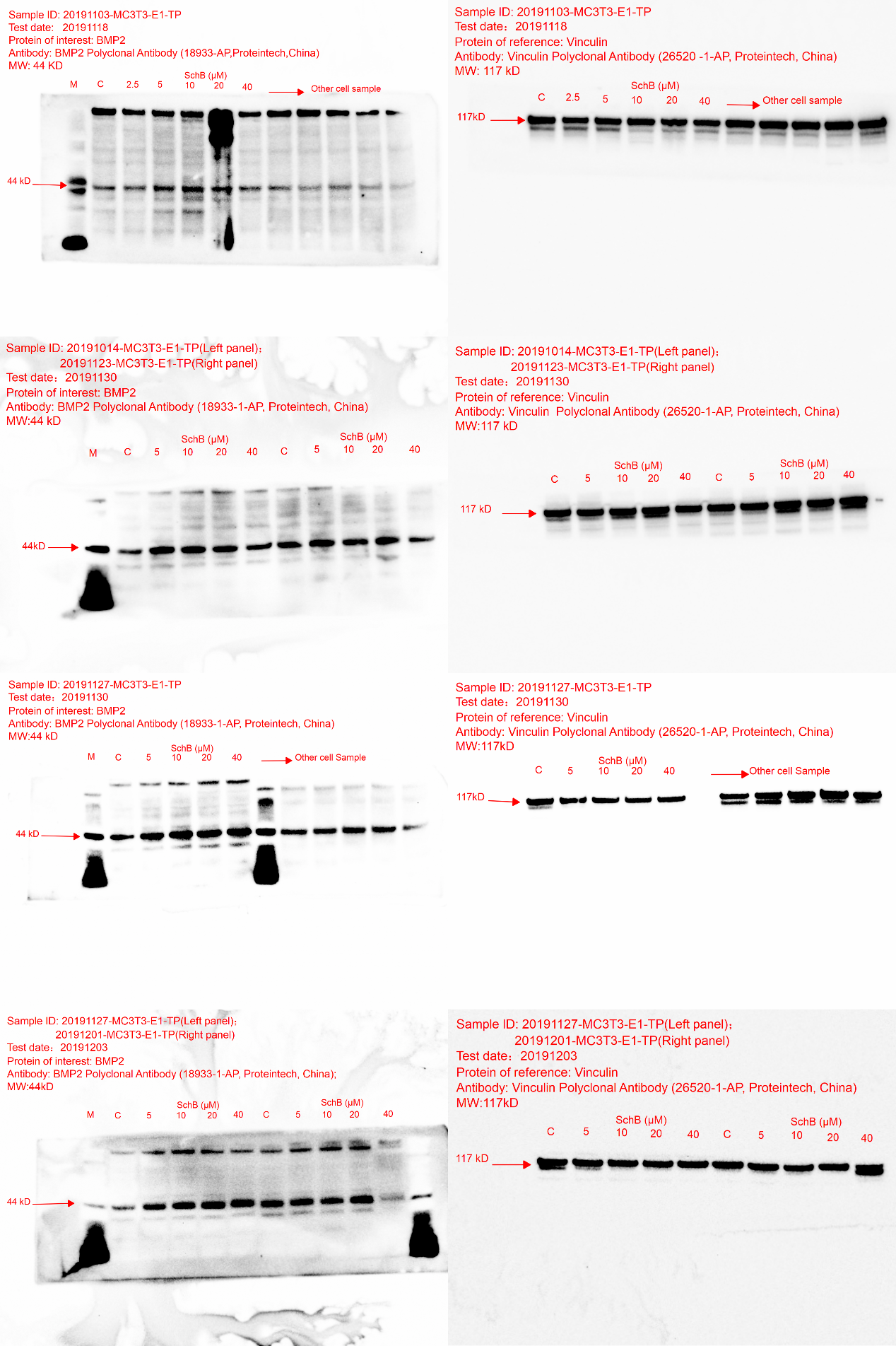


Sup.Fig.1 Original western blots images of BMP2.

2.Fig.3C&D in our manuscript represents the analysis outcomes of the whole uncropped images in Sup.Fig.2.


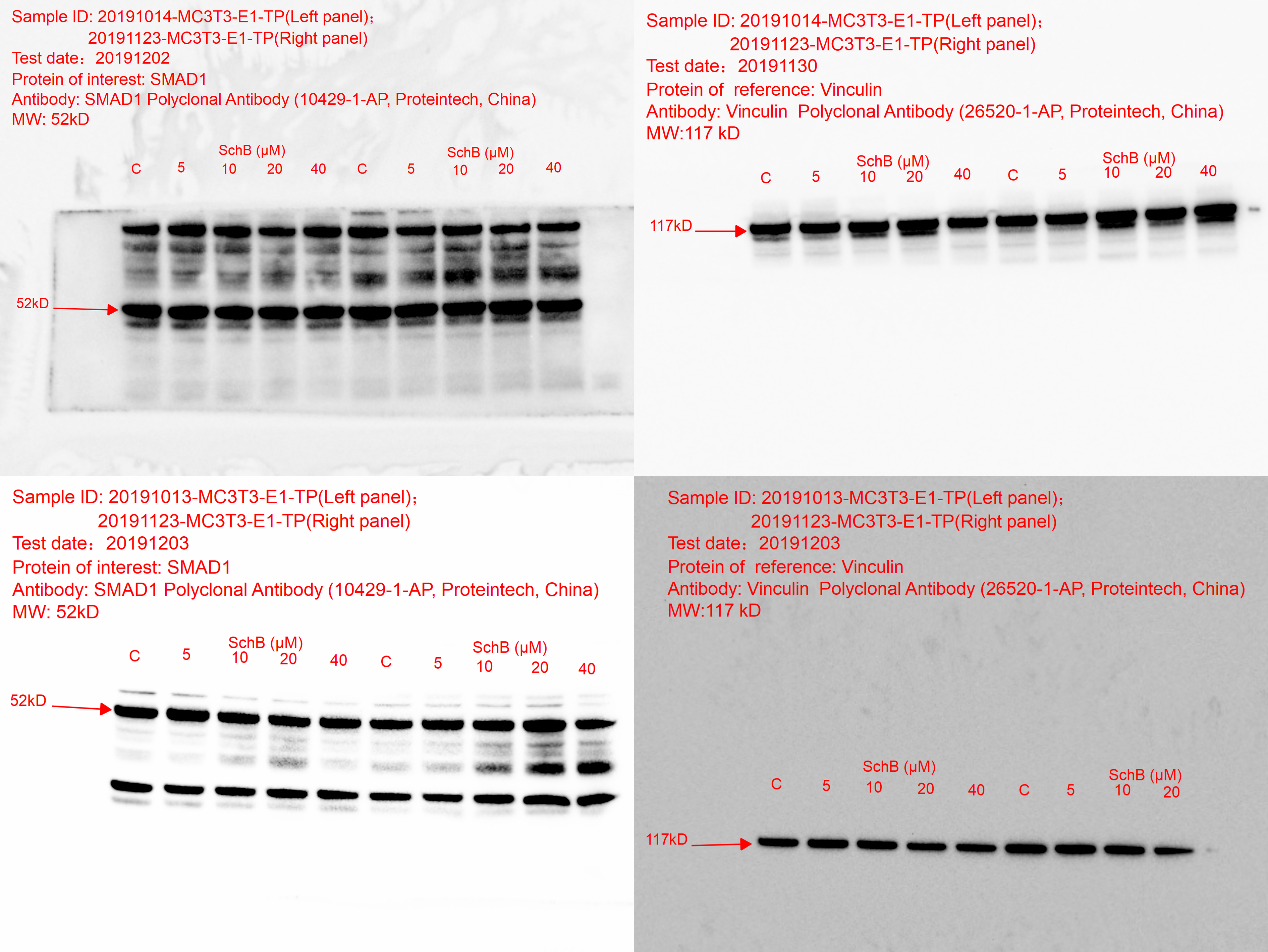


Sup.Fig.2 Original western blots images of SMAD1.

3.Fig.3E&F in our manuscript represents the analysis outcomes of the whole uncropped images in Sup.Fig.3.


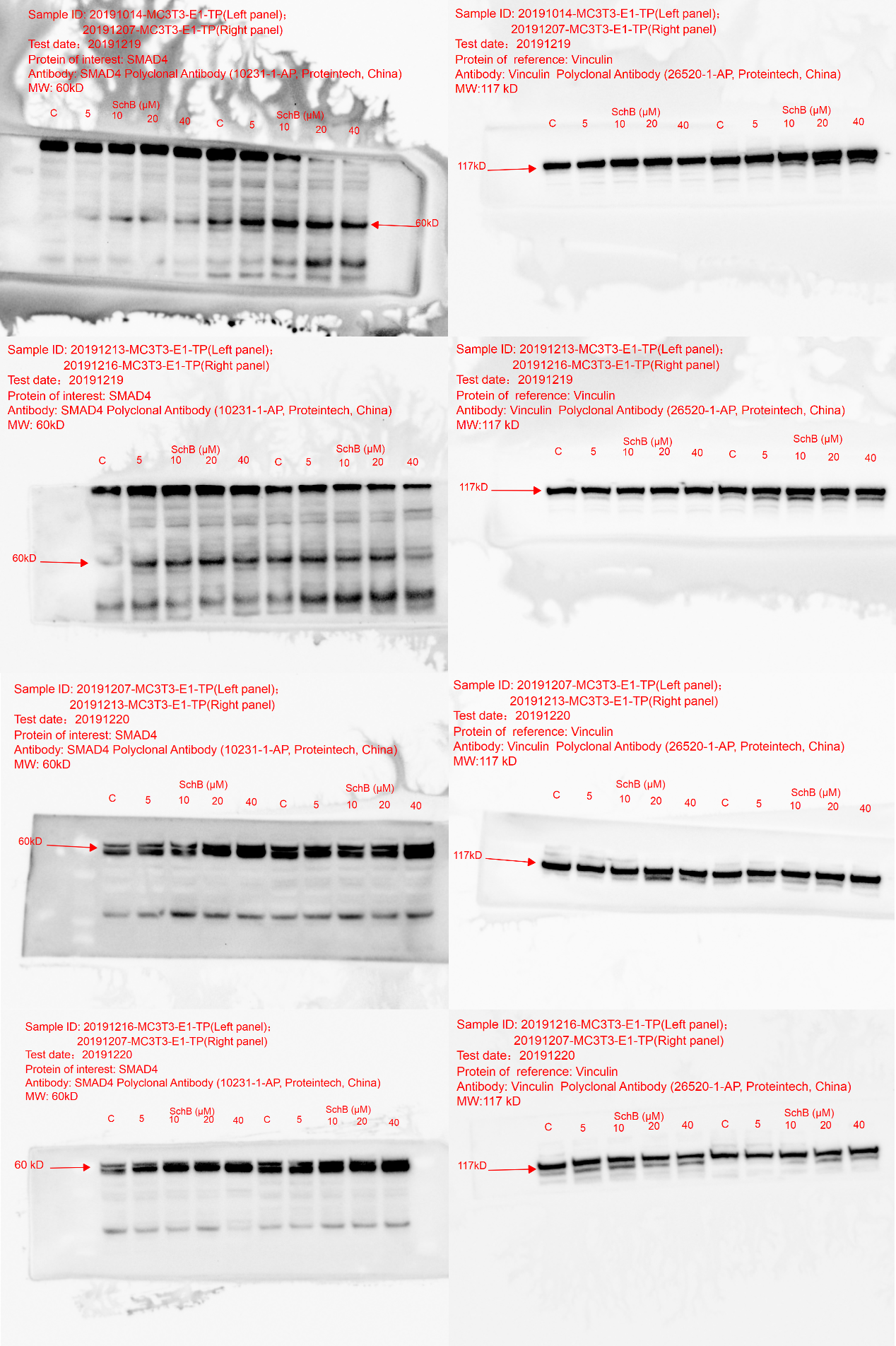


Sup.Fig.3 Original western blots images of SMAD4.

4.Fig.3G&H in our manuscript represents the analysis outcomes of the whole uncropped images in Sup.Fig.4.


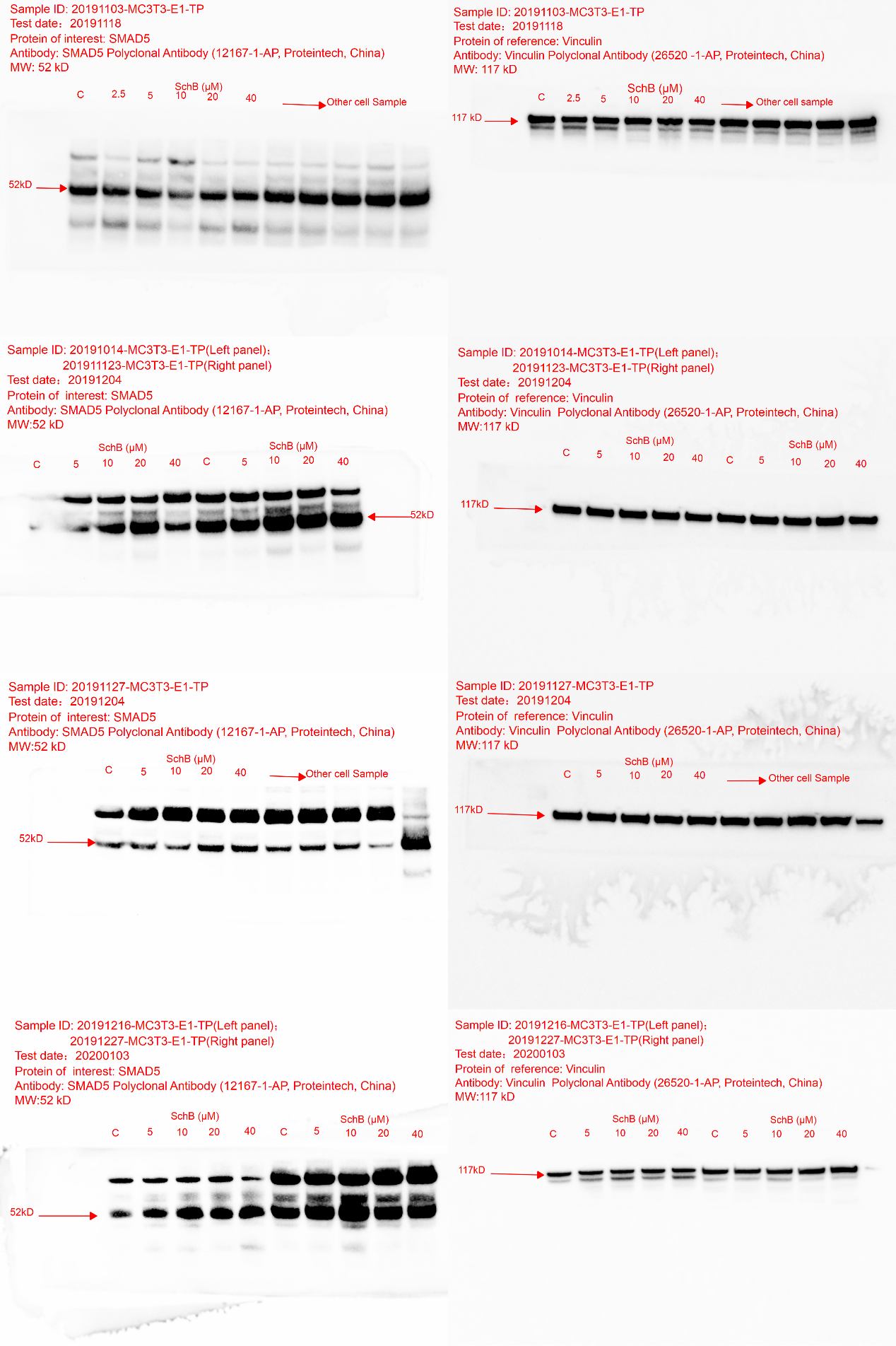


Sup.Fig.4 Original western blots images of SMAD5.

5.Fig.3I&J in our manuscript represents the analysis outcomes of the whole uncropped images in Sup.Fig.5.


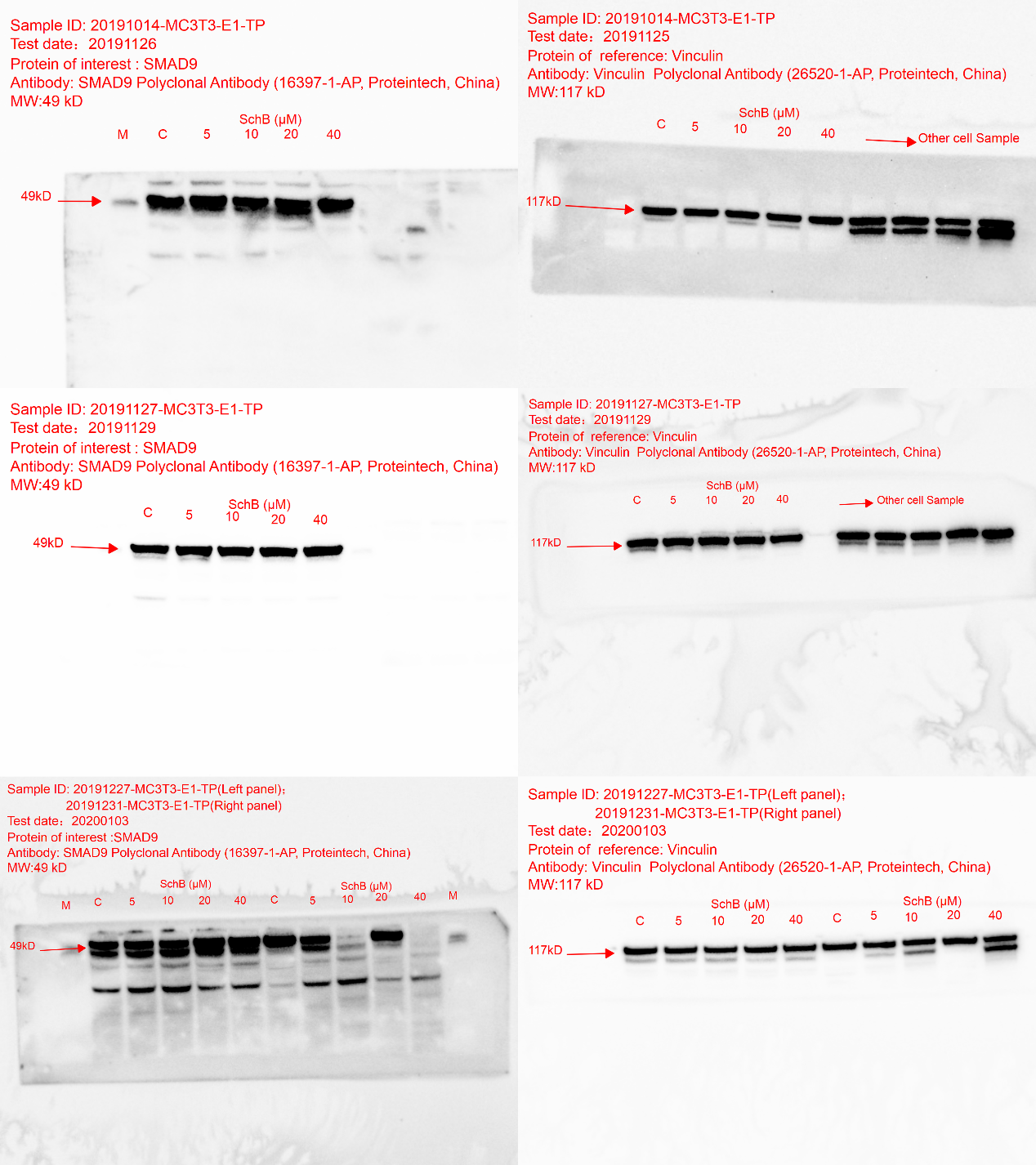


Sup.Fig.5 Original western blots images of SMAD9.

6.Fig.3K&L in our manuscript represents the analysis outcomes of the whole uncropped images in Sup.Fig.6.


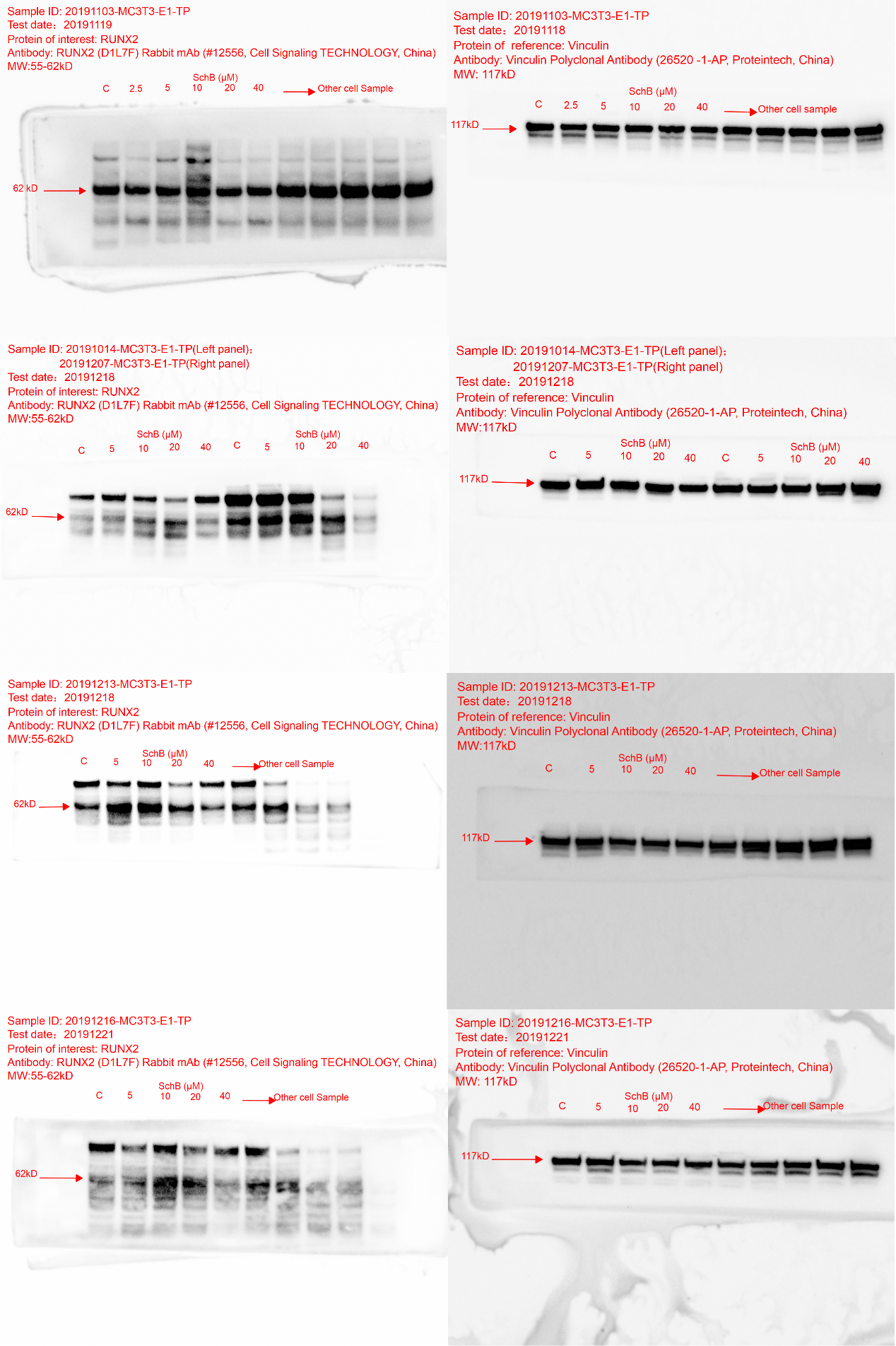


Sup.Fig.6 Original western blots images of RUNX2.

7.Fig.4E, F in our manuscript represents the analysis outcomes of the whole uncropped images in Sup.Fig.7.


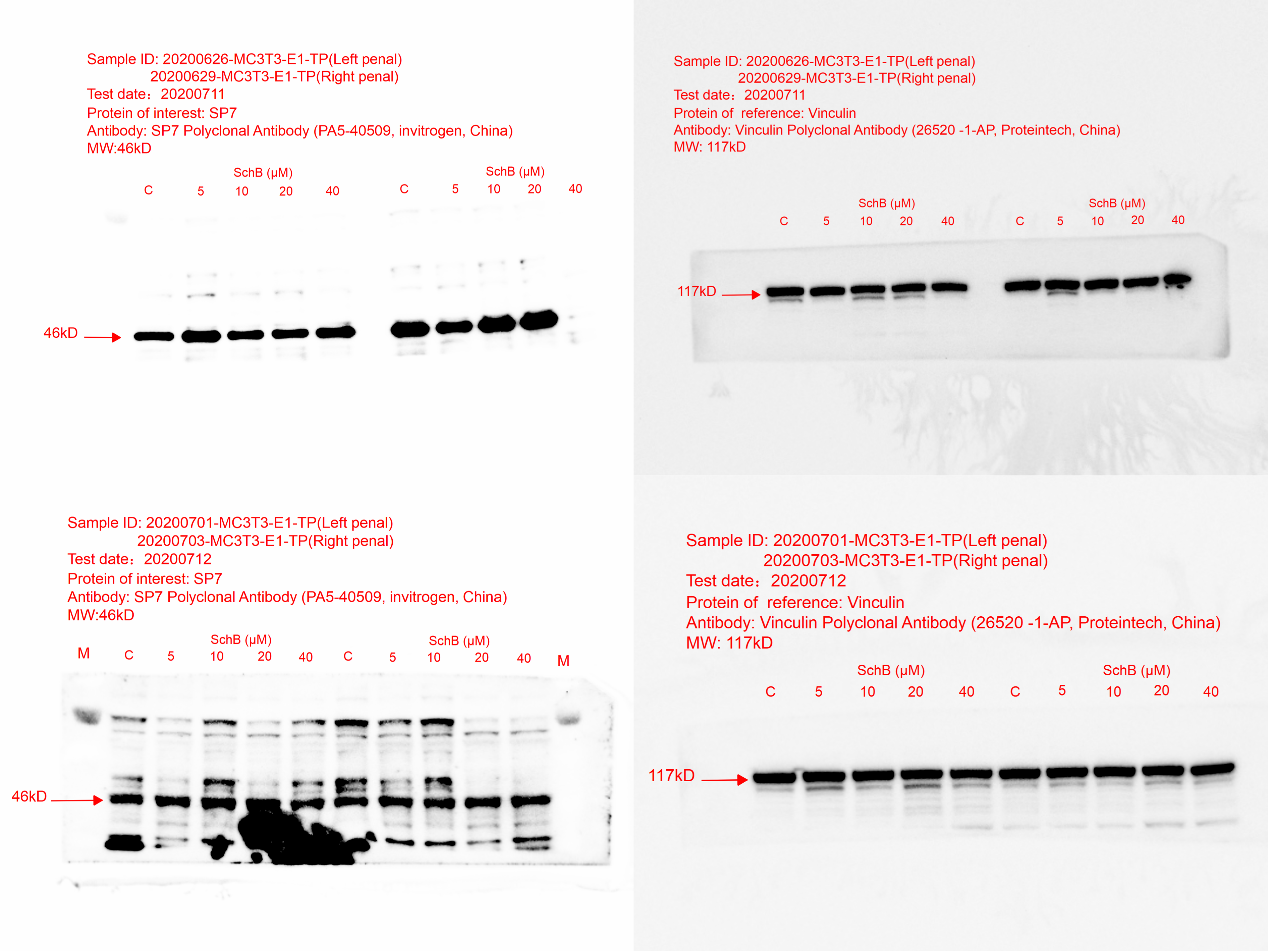


Sup.Fig.7 Original western blots images of Sp7.
